# Cooperative and alternate functions for STIM1 and STIM2 in macrophage activation and in the context of inflammation

**DOI:** 10.1002/iid3.56

**Published:** 2015-05-12

**Authors:** Georgios Sogkas, David Stegner, Shahzad N Syed, Timo Vögtle, Eduard Rau, Britta Gewecke, Reinhold E Schmidt, Bernhard Nieswandt, Johannes Engelbert Gessner

**Affiliations:** 1 Clinical Department of Immunology and Rheumatology, Hannover Medical SchoolGermany; 2 Chair of Experimental Biomedicine University Hospital and Rudolf Virchow Center DFG Research Center for Experimental Biomedicine, University of WürzburgWürzburg, Germany

**Keywords:** Calcium, complement, Fcγ receptors, inflammation, macrophage

## Abstract

Calcium (Ca^2+^) signaling in immune cells, including macrophages, controls a wide range of effector functions that are critical for host defense and contribute to inflammation and autoimmune diseases. However, receptor-mediated Ca^2+^ responses consist of complex mechanisms that make it difficult to identify the pathogenesis and develop therapy. Previous studies have revealed the importance of the Ca^2+^ sensor STIM1 and store-operated Ca^2+^-entry (SOCE) for Fcγ-receptor activation and IgG-induced inflammation. Here, we identify the closely related STIM2 as mediator of cell migration and cytokine production downstream of GPCR and TLR4 activation in macrophages and show that mice lacking STIM2 are partially resistant to inflammatory responses in peritonitis and LPS-induced inflammation. Interestingly, STIM2 modulates the migratory behavior of macrophages independent from STIM1 and without a strict requirement for Ca^2+^ influx. While STIM2 also contributes in part to FcγR activation, the C5a-induced amplification of IgG-mediated phagocytosis is mainly dependent on STIM1. Blockade of STIM-related functions limits mortality in experimental models of AIHA and LPS-sepsis in normal mice. These results suggest benefits of Ca^2+^-inhibition for suppression of exacerbated immune reactions and illustrate the significance of alternate functions of STIM proteins in macrophage activation and in the context of innate immune inflammation.

## Introduction

Several lines of evidence suggest the critical role of macrophages in the maintenance of tissue homeostasis and their contribution in various inflammatory and autoimmune diseases 1–5. Expression of distinct classes of immune receptors, such as FcRs, complement receptors (e.g., C5a anaphylatoxin receptor, C5aR), and TLRs ensures macrophages to respond to diverse stimuli of normal, infectious, and autoimmune origin 6–9. For example, LPS activates TLR4 resulting in the activation of transcriptional mediators including NF-κB, which orchestrate the production of proinflammatory factors 10. C5aR-regulated activation of FcγRs (especially of FcγRIII; 11) together with C5aR- and other chemokine receptor-mediated responses are critical for autoantibody-induced cellular destruction and recruitment of inflammatory cells 12–14.

Macrophage activation can assume a pathogenic role, which may necessitate activation via distinct receptors. The pathogenic relevance of the cross-talk between macrophage C5aR and activating FcγRs has been demonstrated in several animal models of disease, such as autoimmune hemolytic anemia (AIHA), anti-glomerular basement membrane nephritis, and immune complex (IC)-induced tissue injury 9,14–16. Excessive production of TNFα and other toxic cytokines downstream of TLR4 activation can cause septic shock 10,17. Moreover, evidence on direct or indirect interactions between FcγRs and TLR4 has been provided 18,19. Little is known about the pathways that directly impact C5aR, FcγR, and TLR signaling and their relevance in immunological diseases.

Ca^2+^ is a crucial second messenger involved in signaling downstream of several classes of macrophage receptors, including C5aR, FcγRs, and TLRs 20–23. Signaling cascades involving phospholipase C activation result in inositol 1,4,5-triphosphate (InsP_3_)-mediated release of Ca^2+^ from the endoplasmic reticulum (ER) through InsP_3_ receptor channels yielding a rapid but transient increase of cytosolic Ca^2+^ 24–26. ER Ca^2+^ store depletion can induce the opening of plasma membrane-expressed store-operated Ca^2+^ (SOC) channels, also known as calcium release-activated Ca^2+^ (CRAC) channels, resulting in more sustained Ca^2+^ signals 27,28. Such a mode of store-operated Ca^2+^entry (SOCE) is regulated by the ER-resident Ca^2+^ sensor stromal interaction molecule 1 (STIM1) in many cells including mast cells, T and B cells, as well as platelets 29,30. The closely related STIM2 has been also suggested to activate SOC influx, but appears less effective than STIM1 in T and B cells 31–33. Furthermore, STIM2 may have an additional distinct role in regulating basal cytosolic and ER Ca^2+^ concentrations 34,35. The relevance of STIM2 for Ca^2+^-induced activation of macrophages, however, has not been addressed so far, and the effect of STIM proteins on the regulation of major macrophage effector functions and their contribution to disease pathogenesis is not fully understood.

In the present study, we investigated the requirement for STIM2 in macrophage activation and inflammation. Our data suggest a previously unidentified function of STIM2 for TLR-activated cytokine production and GPCR-mediated cell migration independent from STIM1. STIM2, like STIM1, also contributes to SOCE and phagocytosis, but only STIM1 is essential for C5aR-mediated amplification of FcγR activation in vitro and in a model of AIHA in vivo. Thus, STIM1 and STIM2 are critical but distinctly acting mediators of different macrophage effector functions and, as shown for septic shock and fatal hemolysis, inhibition of Ca^2+^ signaling may be beneficial in the treatment of severe inflammation and autoimmune injury.

## Results

### Differential requirement of extracellular Ca^2+^ for phagocytosis, chemotaxis, and TLR4-mediated activation of macrophages

**Figure 1:**
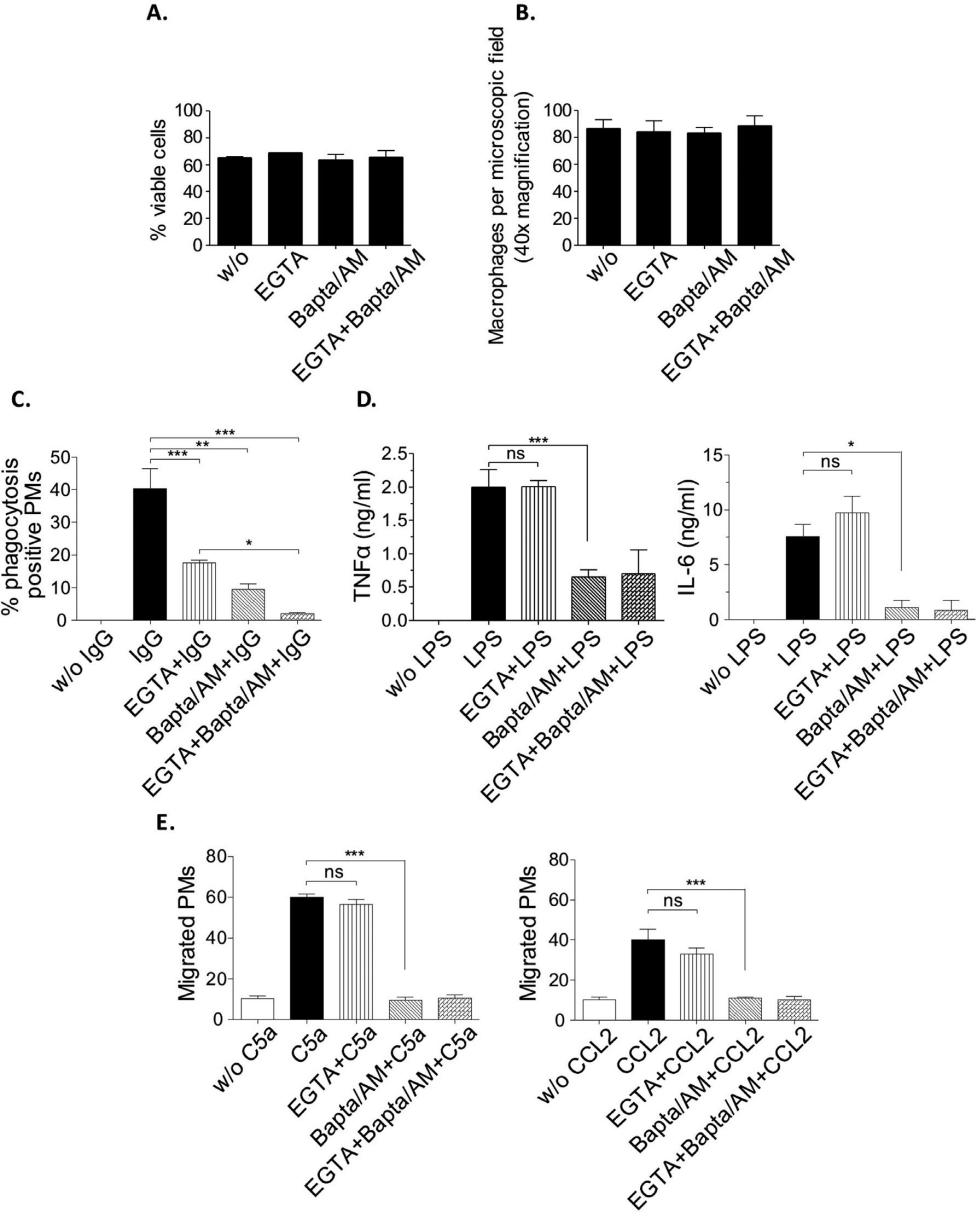
Requirement of extracellular Ca^2+^ for macrophage effector responses of IgG-induced phagocytosis, but not cytokine production and chemotaxis. PM cells from C57Bl/6 mice were cultured in the presence of Ca^2+^-specific chelators (EGTA, or Bapta/AM) or a combination of both (EGTA + Bapta/AM) and assayed for (A) viability, (B) adherence, (C) IgG-induced phagocytosis (IgG), (D) LPS-induced cytokine production (TNFα, IL-6; LPS), and (E) C5a and CCL2-induced chemotactic migration (C5a, CCL2). (C) PM cells were incubated with uncoated (w/o IgG) or IgG-coated (IgG) MRBC for 4 h at 37°C followed by the lysis of extracellular MRBC. The percentage of positive PM cells that ingested more than one MRBC was assessed microscopically. (D) PM cells were incubated for 24 h with (LPS) or without (w/o LPS) 100 ng/mL of LPS and analyzed for production of TNFα and IL-6 by ELISA. (E) C5a (50 ng/mL)- and CCL2 (100 ng/mL)-induced chemotaxis was determined by Transwell migration assays. The number of migrated PM cells detected per microscopic field at 40× magnification is shown. All results are expressed as mean (± SEM) of at least 5 independent experiments (ns, not significant; *P < 0.05; **P < 0.01; ***P < 0.001).

To assess the relative contribution of different sources of Ca^2+^ in key aspects of macrophage biology, we used freshly isolated PMs from C57Bl/6 mice in the presence of the two Ca^2+^-specific chelators EGTA and Bapta/AM. Bapta/AM removes intracellular Ca^2+^, whereas EGTA blocks the influx of extracellular Ca^2+^. Incubation with Bapta/AM (50 μM) and EGTA (2 mM) had no effect on the viability (Fig. 1A) and adhesive capacity (Fig. 1B) of PM cells. We first investigated the effects of the two Ca^2+^ chelators on FcγR-dependent phagocytosis. PM cells were incubated with MRBCs opsonized with anti-MRBC IgG and measured for phagocytosis of MRBCs by light microscopy as described 36. Macrophages showed strongly diminished IgG-mediated phagocytosis upon removal of either extracellular Ca^2+^ (by EGTA) or intracellular Ca^2+^ (by Bapta/AM) (Fig. 1C). Simultaneous application of EGTA and Bapta/AM completely abolished phagocytosis, providing evidence that FcγR-mediated phagocytosis requires sustained mobilization of Ca^2+^ from both the intracellular stores and the extracellular milieu. We also examined LPS-induced cytokine production. Strikingly, similar levels of TNFα and IL-6 were detected in the presence and absence of EGTA in cell-culture supernatant of PMs stimulated with LPS, whereas inhibition of intracellular Ca^2+^ by Bapta/AM resulted in reduced secretion of TNFα and IL-6 (Fig. 1D). Next, we tested the role of Ca^2+^ for the migratory capacity of PMs in response to CCL2 and C5a in standard Transwell chemotaxis assays. Similar to TLR4-mediated activation and the expression of cytokines, inhibition of intracellular Ca^2+^ (but not of Ca^2+^ influx) yielded substantially decreased chemotaxis (Fig. 1E). Taken together, these results indicate that FcγR-mediated phagocytosis, but not TLR4-mediated activation or CCL2-/C5a-induced migration is dependent on extracellular Ca^2+^ in macrophages.

### STIM1 is essential for C5a-induced upregulation of FcγR-mediated phagocytosis but not for cytokine production and macrophage migration in vitro and in vivo

**Figure 2:**
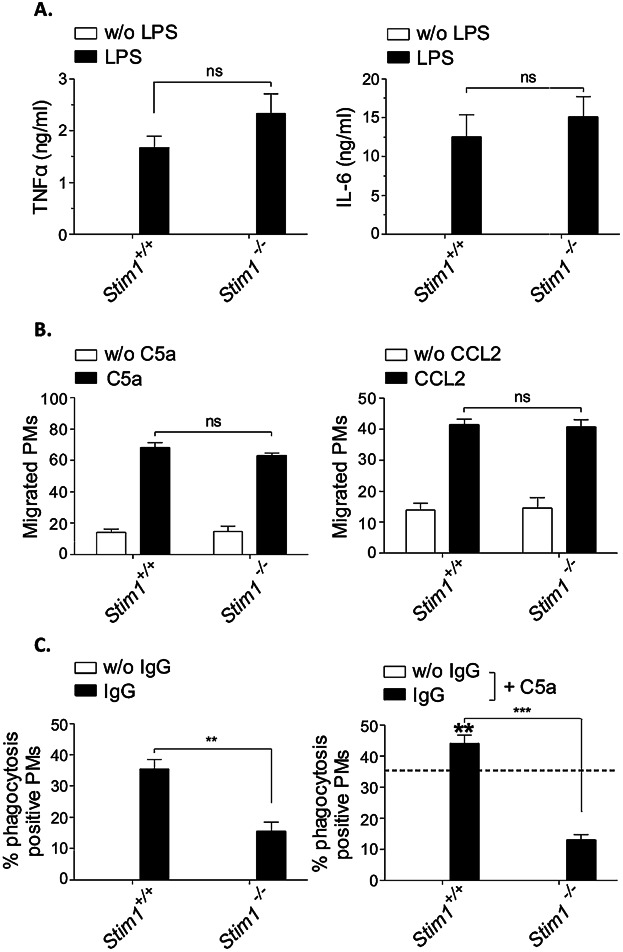
Normal cytokine release and chemotaxis, but defective IgG-induced and C5a-regulated phagocytosis in *Stim1*^−/−^ macrophages. (A) PM cells of *Stim1*^−/−^ and *Stim^+/+^* chimeric mice were stimulated with 100 ng/mL LPS and analyzed at 24 h for the release of TNFα and IL-6 by ELISA. (B) PMs were stimulated with C5a and CCL2 for chemotaxis in Transwell migration assays, and migrated cells were counted under light microscopy. (C) PM cells were incubated with uncoated (w/o IgG) or IgG-coated MRBCs and percentage of phagocytosis was assessed (left panel). Phagocytosis of IgG-opsonized MRBCs by *Stim1^+/+^* PMs (indicated by the intermittent line) was increased in the presence of 50 ng/mL of C5a (IgG + C5a; significance is shown in bold) in *Stim1^+/+^* but not *Stim1*^−/−^ cells (right panel). All results are expressed as mean ± SEM of 3–4 independent experiments (ns, not significant; **P < 0.01; ***P < 0.001). *Stim1*^−/−^ and *Stim1^+/+^* PM cells only differed significantly for IgG-induced and C5a-regulated phagocytosis.

We have previously shown that FcγR-mediated phagocytosis and IgG IC-inflammation are mediated by STIM1-dependent mechanisms and that STIM1 is an essential regulator of SOCE in PMs 37. The role of STIM1 in other Ca^2+^-dependent, but EGTA-insensitive (Ca^2+^ influx-independent) responses of macrophages, however, has not been addressed by these previous studies. Here, we induced TLR4-mediated cytokine production and agonist (CCL2/C5a)-induced migration in STIM1-deficient PM cells in vitro. Similar to the EGTA results obtained before (see Fig. 1), neither LPS-induced secretion of TNFα and IL-6 (Fig. 2A) nor CCL2/C5a-induced chemotaxis (Fig. 2B) was reduced in *Stim1*^−/−^ PMs compared with WT controls. Importantly, FcγR-mediated phagocytosis and its further enhancement by C5a were almost completely absent in *Stim1*^−/−^ macrophages (Fig. 2C), demonstrating that STIM1—although not necessary for chemotaxis and TLR4-mediated cytokine production—is essential for phagocytosis and its C5a-induced regulation.

**Figure 3:**
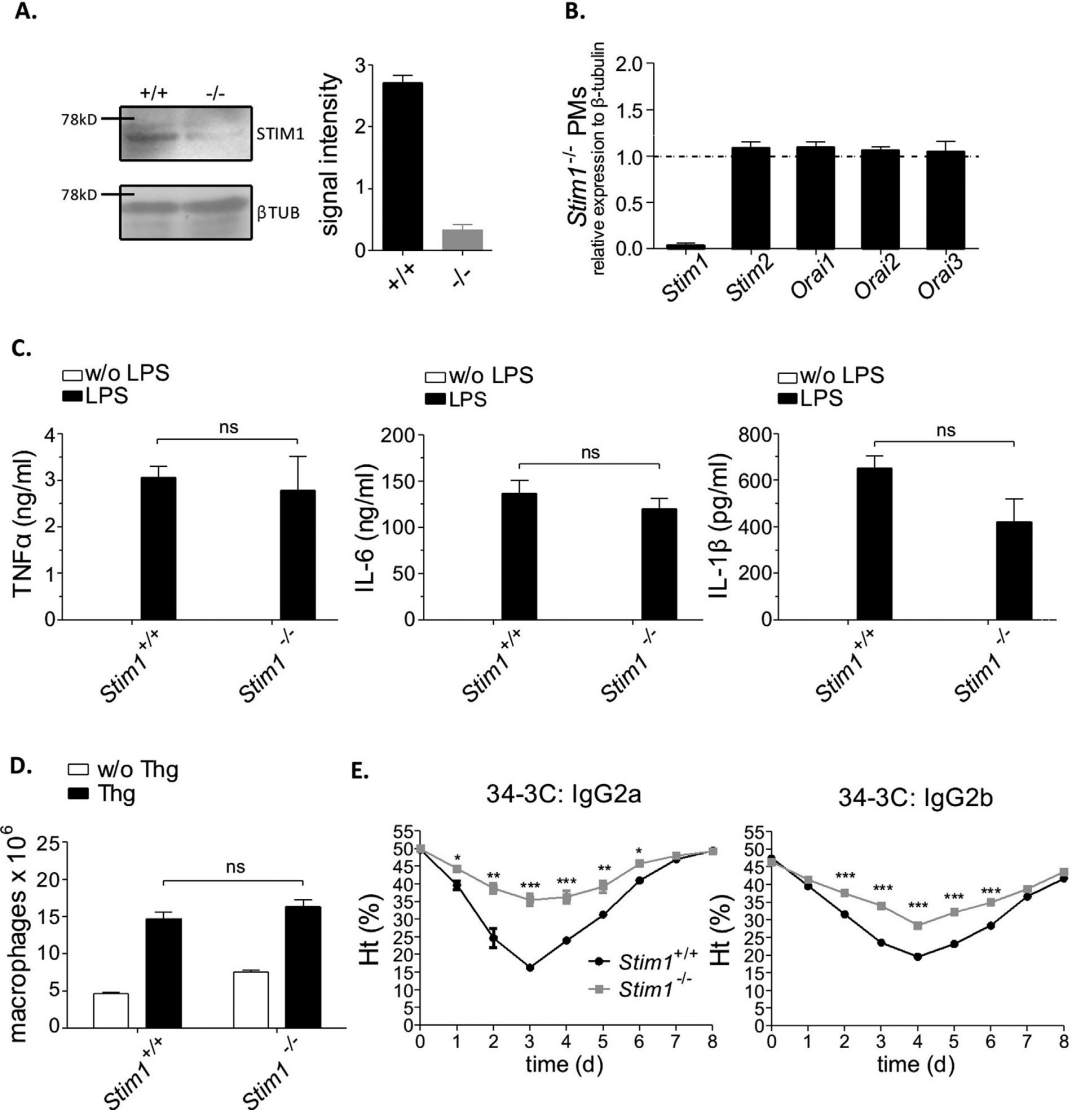
*Stim1*^−/−^ chimeric mice show normal signs of LPS- and Thg-induced inflammation but reduced pathology in IgG-induced AIHA. (A) Western blot analysis of STIM1 expression in *Stim1*^−/−^ and *Stim1^+/+^* chimeric mice; β-tubulin (βTUB) served as a loading control. Left panel: representative blot. Right panel: densitometric analysis of three individual blots. The results shown are the mean signal intensities (±SEM) for STIM1, each normalized to the signal intensity of the β-tubulin signal of the same sample (arbitrarily defined as 1.0). (B) RT-qPCR analysis of Orai1, Orai2, Orai3, STIM1, and STIM2 in *Stim1*^−/−^ PM cells (the dashed line indicates wild-type expression in *Stim1^+/+^* PM cells). Data were analyzed using the *δ*–*δ* Ct method. (C) Inflammation was induced by i.p. injection of 10 mg/kg LPS in *Stim1*^−/−^ and *Stim1^+/+^* chimeric mice. After 2 h, sera were measured for production of TNFα, IL-6, and IL-1β by ELISA. (D) Mice received 4% Thg i.p. and recruitment of macrophages into peritoneum was analyzed at day 4. (E) AIHA was induced by injection of 150 μg of the pathogenic anti-MRBC 34-3C IgG2a and IgG2b mAbs, and daily hematocrit Ht (%) was assessed for 8 days. The results shown are mean ± SEM of four to six mice per group (*P < 0.05; **P < 0.01; ***P < 0.001).

To further corroborate the in vitro findings we subjected *Stim1*^−/−^ bone marrow chimeric mice to different in vivo models of LPS-induced sepsis, Thg-induced peritonitis, and AIHA. Immunoblot analysis and RT-qPCR confirmed the absence of STIM1 in PM preparations from the mutant chimeras, whereas the mRNA and protein were strongly expressed in control PM (Fig. 3A, B). Furthermore, the mRNA levels of *Stim2* and Orai isoforms (*Orai1*, *Orai2*, and *Orai3*) were almost unaltered in *Stim1*^−/−^ PM cells (Fig. 3B). A hallmark of the early response in LPS-induced acute inflammation is the massive production of proinflammatory cytokines, such as TNFα, IL-6, and IL-1β 38. Serum levels of these cytokines were studied at 2 h after i.p. injection with LPS (10 mg/ kg). No difference was detected between *Stim1*^−/−^ chimeric mice and WT controls (Fig. 3C). In the second model, Thg-induced cell recruitment into the peritoneal cavity was examined on day 4 by counting macrophage numbers obtained after peritoneal lavage. Again, *Stim1*^−/−^ and WT chimeras each showed normal macrophage elicitation (Fig. 3D). In the model of AIHA 15,36, however, STIM1 deficiency provides reduction from anemia induced by pathogenic anti-MRBC 34-3C autoantibodies of IgG2a and IgG2b subclasses (Fig. 3E).

### STIM2 contributes to macrophage SOCE, FcγR-mediated Ca^2+^, and phagocytosis but is not required for C5a-induced regulation of FcγR-dependent MRBC cell destruction and AIHA

**Figure 4:**
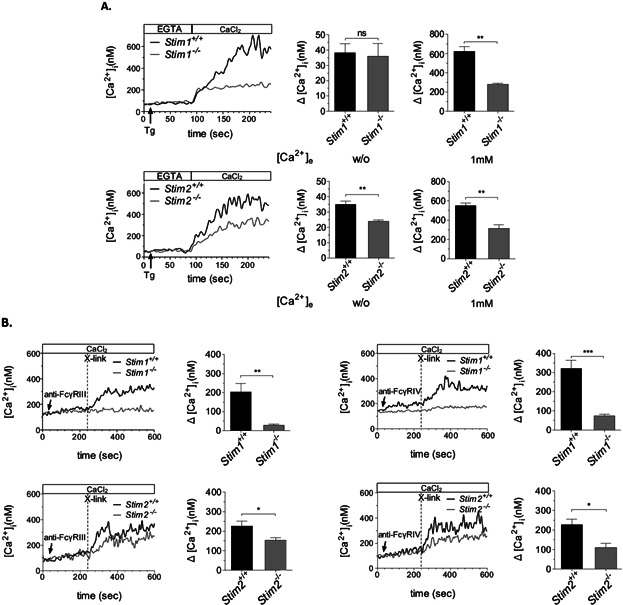
Distinct and quantitatively different defects of TG-induced Ca^2+^ ER store release, SOCE, and FcγR-mediated Ca^2+^ in *Stim1*^−/−^ and *Stim2*^−/−^ macrophages. (A) *Stim1*^−/−^ and *Stim2*^−/−^ PM cells were loaded with Fura2/AM, stimulated with 2 μM TG in EGTA-containing buffer (w/o [Ca^2+^]_e_) followed by the addition of CaCl_2_ (1 mM [Ca^2+^]_e_) and monitoring of [Ca^2+^]_i_. Representative measurements (left panels) and maximal (Δ[Ca^2+^]_i_ ± SEM) values (*n* = 5–7 per group) in the absence (w/o [Ca^2+^]_e_) (middle panels) and presence (1 mM [Ca^2+^]_e_) of extracellular Ca^2+^ (right panels) are shown (**P < 0.01). (B) Fura2/AM-loaded PMs were incubated with 10 μg/mL anti-FcγRIII and anti-FcγRIV mAbs in the presence of 1 mM of extracellular Ca^2+^ (CaCl_2_) followed by the addition of 20 μg/mL of cross-linking antibodies (x-link; dashed line) and monitoring of [Ca^2+^]_i_. Representative measurements and maximal (Δ[Ca^2+^]_i_ ± SEM) values (*n* = 3–4 per group) (left: FcγRIII-mediated Ca^2+^; right: FcγRIV-mediated Ca^2+^) are shown (*P < 0.05; **P < 0.01; ***P < 0.001).

Analysis of STIM2-deficient mice has recently suggested that STIM2 may co-activate SOCE in T and B cells 32,33. In contrast, the importance of STIM2 in relation to STIM1 for SOCE and FcγR-mediated Ca^2+^ mobilization in macrophages remained unknown. Thus, we compared the roles of STIM1 and STIM2 for SOCE. SOC influx in *Stim1*^−/−^, *Stim2*^−/−^, and matched WT PMs was triggered by stimulating them with thapsigargin. TG-induced Ca^2+^ store release was reduced approximately 30% in *Stim2*^−/−^ PMs compared with wild-type controls (Fig. 4A). Subsequent TG-dependent SOC influx, however, was substantially more decreased in *Stim1*^−/−^ compared to *Stim2*^−/−^ PMs. We also examined the capacity of STIM-deficient macrophages to respond to FcγR activation. *Stim2*^−/−^ PMs showed only a modest reduction in the FcγRIII- and FcγRIV-mediated elevation of Ca^2+^, whereas cytosolic Ca^2+^ levels were much lower when STIM1 was absent (Fig. 4B). These results show that both STIM1 and (to a lesser extent) STIM2 contribute to SOCE and FcγR-mediated Ca^2+^ responses and indicate that STIM2 is also required in part for proper control of store content in macrophages.

**Figure 5:**
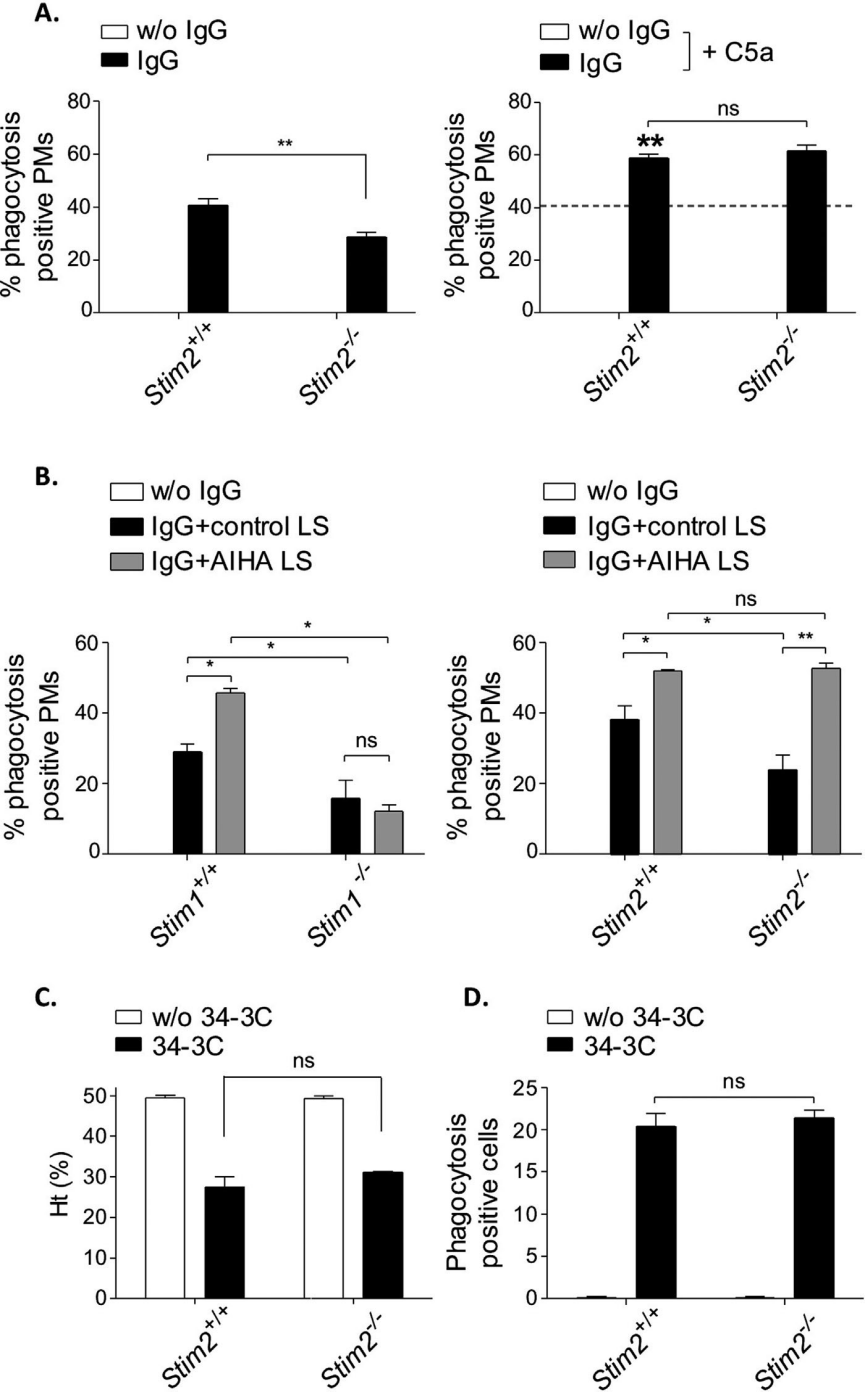
Normal C5a-regulated phagocytosis and AIHA in *Stim2*^−/−^ mice. (A) *Stim2*^−/−^ and *Stim2^+/+^* PM cells were incubated with uncoated (w/o IgG) or IgG-coated MRBCs and percentage of phagocytosis was assessed (left panel). Phagocytosis of IgG-opsonized MRBCs by *Stim2^+/+^* PMs (indicated by the intermittent line) was increased in the presence of 50 ng/mL of C5a (IgG + C5a; significance is shown in bold) in both *Stim2^+/+^* and *Stim2*^−/−^ cells (right panel). The results are expressed as mean ± SEM of three to four independent experiments (**P < 0.01). (B) *Stim1*^−/−^ and *Stim2*^−/−^ PM cells and their matched (*Stim1^+/+^* and *Stim2^+/+^*) WT controls were assayed for enhancement of phagocytosis by liver supernatant from control mice (control LS), or from AIHA-induced C57Bl/6 mice (AIHA LS). The results are expressed as mean ± SEM of three to four independent experiments (*P < 0.05; **P < 0.01). Note the significantly induced phagocytosis by C5a-containing AIHA LS in *Stim2*^−/−^ but not *Stim1*^−/−^ PMs. (C, D) Experimental AIHA was induced by 150 μg of the anti-MRBC 34-3C IgG2a mAb in *Stim2*^−/−^ and *Stim2^+/+^* mice. The results are shown as the mean ± SEM of (C) hematocrit and (D) liver phagocytosis at days 0 and 2 from 5 mice per group (ns, not significant).

To determine the consequences of STIM2 deficiency for FcγR-mediated macrophage effector functions, we then studied erythrophagocytosis and its associated C5a-induced regulation both in vitro and in vivo. *Stim2*^−/−^ PMs showed reduced IgG-induced phagocytosis. This defect, however, could be rescued and phagocytosis efficiency could be increased by the addition of C5a (Fig. 5A). Interestingly and in contrast to the case of STIM1 deficiency, macrophage exposure to liver supernatant derived from AIHA-subjected B6 mice—previously reported to contain C5a 14—also reinforced defective phagocytosis in *Stim2*^−/−^ PMs (Fig. 5B). This suggests that STIM2—while contributing to some extent to phagocytosis in vitro—may not play a critical role in AIHA. Indeed, STIM2-deficient mice showed no negative effect on 34-3C mAb-induced anemia in the clearance of MRBC as indicated by Ht levels (Fig. 5C) and the percentage of liver cells containing ingested erythrocytes (Fig. 5D).

### STIM2 is selectively required for effective CCL2- and C5a-induced macrophage migration and TLR4-mediated cytokine production in vitro and in vivo

**Figure 6:**
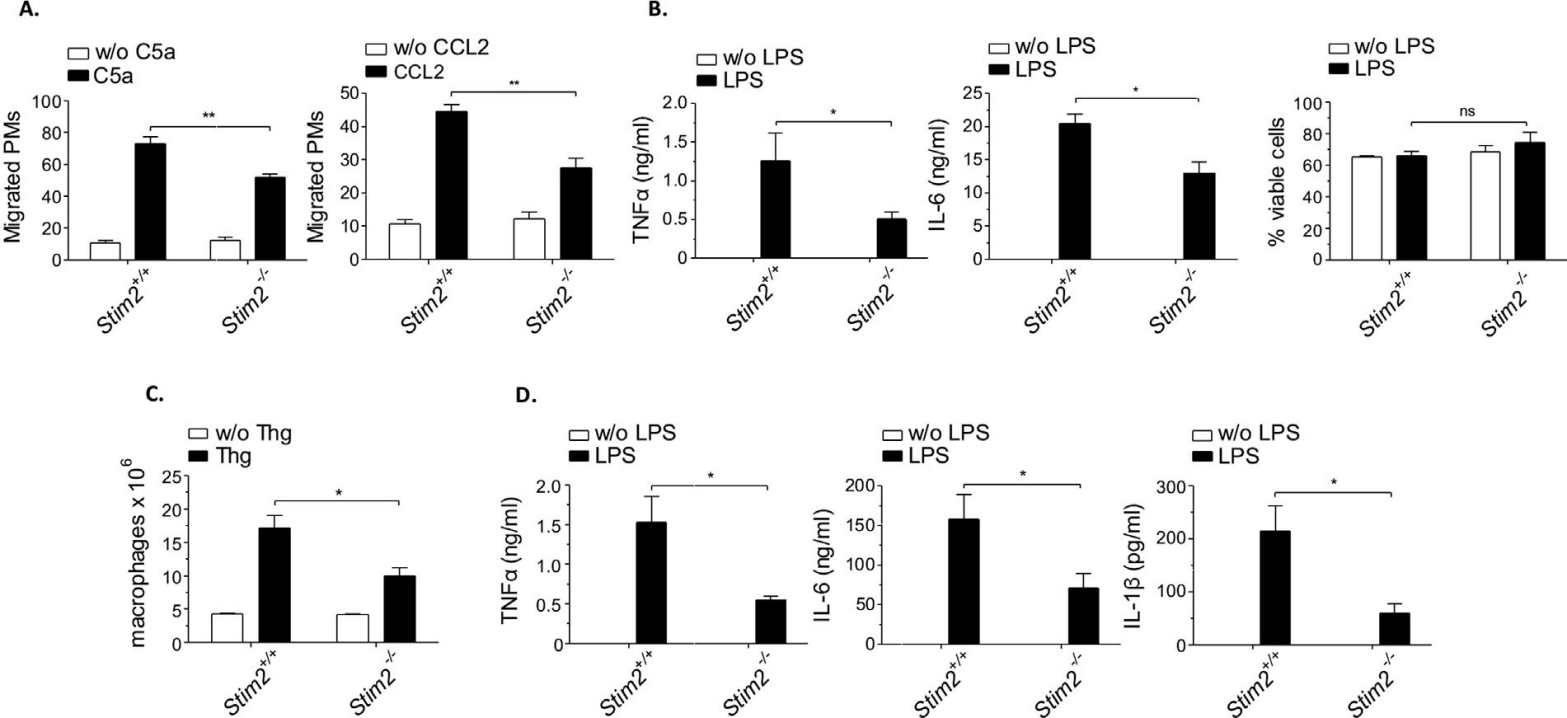
Requirement of STIM2 for macrophage chemotaxis and cytokine release in vitro, and Thg- and LPS-stimulated inflammation in vivo. (A) *Stim2^−/−^* and *Stim2^+/+^* PM cells were assayed for C5a- and CCL2-induced chemotaxis in Transwell migration assays, and migrated cells were counted by light microscopy. (B) PMs were stimulated with 100 ng/mL LPS and analyzed at 24 h for viability (right panel) and the release of TNFα and IL-6 by ELISA (left, middle panel). (A, B) The results are expressed as mean ± SEM of three to four independent experiments (*P < 0.05; **P < 0.01). (C) *Stim2^−/−^* and *Stim2^+/+^* mice received 4% Thg i.p. and recruitment of macrophages into peritoneum was analyzed at day 4. (D) Inflammation was induced in mice by 10 mg/kg LPS i.p. and sera were measured at 2 h for production of TNFα, IL-6, and IL-1β by ELISA. (C, D) The results shown are mean ± SEM of seven mice per group (*P < 0.05).

The above results indicated that both STIM1 and STIM2 contribute to phagocytosis and Ca^2+^ responses downstream of FcγRs. However, only STIM1 plays a critical role in C5a-induced regulation of FcγR-mediated phagocytosis and, as a consequence, in the pathogenesis of AIHA. On the other hand, STIM1 appears not to be involved in EGTA-insensitive macrophage Ca^2+^ responses, as exemplified for CCL2/C5a-induced migration and TLR4-mediated cytokine production. To address the role of STIM2 in these functions, we analyzed STIM2-deficient macrophages in vitro and *Stim2*^−/−^ mice in vivo. In contrast to the results obtained for *Stim1*^−/−^ PMs, CCL2- and C5a-induced chemotaxis (Fig. 6A) and TLR4-induced secretion of TNFα and IL-6 (Fig. 6B) were reduced in *Stim2*^−/−^ PM cells. Furthermore, STIM2-deficient animals showed decreased macrophage recruitment in the model of Thg-induced peritonitis (Fig. 6C) and reduced TNFα, IL-6, and IL-1β serum levels in LPS-induced inflammation (Fig. 6D).

**Figure 7:**
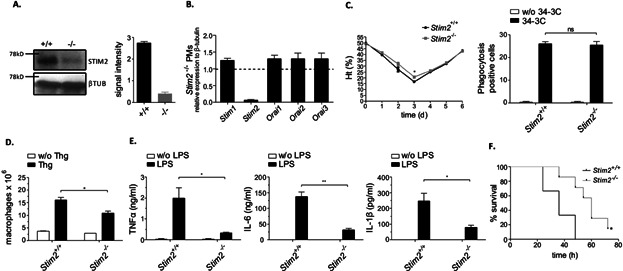
*Stim2*^−/−^ BM chimeric mice show normal pathology in IgG-induced AIHA but reduced signs of Thg- and LPS-induced inflammation. (A) Western blot analysis of STIM2 expression in *Stim2*^−/−^ and *Stim2^+/+^* chimeric mice; β-tubulin (βTUB) served as a loading control. Left panel: representative blot. Right panel: densitometric analysis of three individual blots. (B) RT-qPCR analysis of Orai1, Orai2, Orai3, STIM1, and STIM2 in *Stim2*^−/−^ PM cells (the dashed line indicates wild-type expression in *Stim2^+/+^* PM cells). Data were analyzed using the *δ*–*δ* Ct method. (C) Experimental AIHA was induced by 150 μg of the anti-MRBC 34-3C IgG2a mAb and daily hematocrit Ht (%) was assessed for 6 days. The results are shown as the mean ± SEM of hematocrit and liver phagocytosis (at day 2) from 5 mice per group (ns, not significant). (D) Mice received 4% Thg i.p. and recruitment of macrophages into peritoneum was analyzed at day 4. (E) *Stim2*^−/−^ and *Stim2^+/+^* chimeric mice received 10 mg/kg LPS and serum levels of TNFα, IL-6, and IL-1β were examined at 2 h. The results shown are mean ± SEM of five mice per group (ns; *P < 0.05; **P < 0.01). (F) The indicated mice received a dose of 30 mg/kg of LPS and % survival (*n* = 5 per group) is shown (*P < 0.05).

In order to restrict STIM2 deficiency to the hematopoietic system, we transplanted lethally irradiated wild-type mice with bone marrow from *Stim2*^−/−^ or WT control mice and analyzed them after 10–16 weeks. We confirmed the absence of STIM2 in PM cells from *Stim2*^−/−^ chimeras by Western blot analysis and RT-qPCR (Fig. 7A, B). Transcript levels of STIM1 and Orai1, 2, 3 were almost unaltered in *Stim2*^−/−^ PM cells (Fig. 7B). Similar to STIM2-deficient mice (see Figs. 5, 6), *Stim2*^−/−^ chimeras showed no negative effect on 34-3C mAb-induced AIHA (Fig. 7C) but exhibited reduced Thg-peritonitis (Fig. 7D) and LPS inflammation (Fig. 7E). Moreover, *Stim2*^−/−^ chimeras injected with a lethal dose of LPS showed improved survival (Fig. 7F). The results suggest a role of STIM2 in hematopoietic cells in the control of cell migration and cytokine production that appears of relevance in severe inflammation.

### Silencing of STIM proteins by RNA interference confirm the STIM-isoform specific role of STIM2 for EGTA-insensitive Ca^2+^ responses in macrophage activation

To further strengthen our findings on alternate functions of STIM proteins in calcium activation, we silenced STIM1 and STIM2 in RAW 264.7 macrophages by STIM1 and STIM2 shRNA expression. The obtained KD cells exhibited strongly decreased STIM mRNA and protein levels with no compensatory up- or down-regulation of STIM1 and STIM2 in the absence of the other STIM isoform (Supplemental Fig. S1). In line with the effect of EGTA- or Bapta/AM-mediated Ca^2+^ depletion/chelation in PM cells (Fig. 1), phagocytosis, but not CCL2/C5a-induced migration is dependent on extracellular Ca^2+^ in RAW 264.7 macrophages (Supplemental Fig. S2).

**Figure 8:**
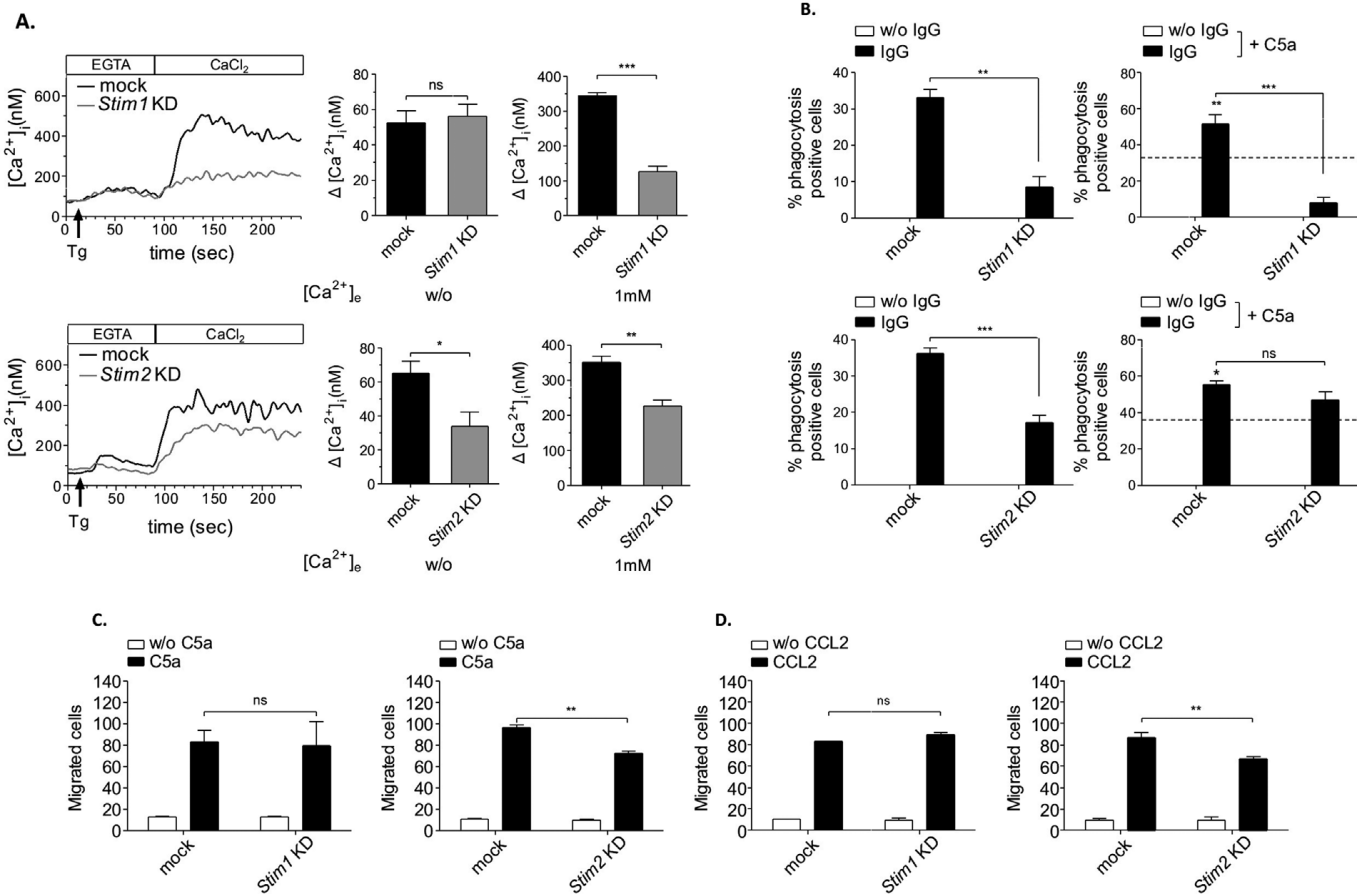
Confirmative evidence for alternate functions of STIM1 and STIM2 that are differentially defective in *Stim1* and *Stim2* KD macrophages. (A) *Stim1* and *Stim2* KD RAW264.7 cells and mock-transfected controls were loaded with Fura2/AM, stimulated with 2 μM TG in EGTA-containing buffer (w/o [Ca^2+^]_e_) followed by the addition of CaCl_2_ (1 mM [Ca^2+^]_e_) and monitoring of [Ca^2+^]_i_. Representative measurements (upper/lower left panels) and maximal (Δ[Ca^2+^]_i_ ± SEM) values (*n* = 5 per group) in the absence (w/o [Ca^2+^]_e_) (upper/lower middle panels) and presence (1 mM [Ca^2+^]_e_) of extracellular Ca^2+^ (upper/lower right panels) are shown (*P < 0.05; **P < 0.01; ***P < 0.001). Only *Stim2* KD cells show a significant defect of TG-induced Ca^2+^ ER store release, whereas SOCE appears much lower in *Stim1* KD cells. (B) The indicated cells were incubated with uncoated (w/o IgG) or IgG-coated MRBCs and percentage of phagocytosis was assessed (upper/lower left panels). Phagocytosis of IgG-opsonized MRBCs by mock-transfected cells (indicated by the intermittent line) was increased in the presence of 50 ng/mL of C5a (IgG + C5a; significance is shown in bold) in *Stim2* but not *Stim1* KD RAW264.7 cells (upper/lower right panels). (C, D) Cells were assayed for C5a- and CCL2-induced chemotaxis in Transwell migration assays, and migrated cells were counted by light microscopy. (B–D) The results are expressed as mean ± SEM of four to five independent experiments (**P < 0.01; ***P < 0.001).

SOCE was detectable in mock transfected RAW 264.7 cells and KD of STIM1 caused a more reduced influx of extracellular Ca^2+^ as compared with *Stim2* KD cells (Fig. 8A). Importantly, the TG-induced Ca^2+^ ER store release was reduced in *Stim2* KD but not *Stim1* KD or mock-transfected cells (Fig. 8A). These findings reveal that STIM1 is most critical for SOCE and confirm the additional function of STIM2 in regulating Ca^2+^ store content. We also evaluated the effects of silenced STIM expression in phagocytosis and chemotaxis. Comparable to the situation of *Stim1*^−/−^ and *Stim2*^−/−^ PMs, C5a-induced regulation of FcγR-mediated phagocytosis is defective in *Stim1* KD cells (Fig. 8B), whereas chemotactic migration induced by C5a (Fig. 8C) and CCL2 (Fig. 8D) was reduced in *Stim2* KD cells, confirming STIM protein selectivity for distinct Ca^2+^-triggered macrophage effector responses.

### The Ca^2+^ flux inhibitor BTP2 further affects STIM2-regulated functions independent from Orai1

We next tested whether the inhibitor BTP2, previously reported to inhibit Ca^2+^ entry in T cells 39,40, blocks STIM1-mediated SOCE and maybe other Ca^2+^ signals in macrophages. Interestingly, we found that BTP2 not only reduced SOCE but also abrogated the STIM2-sensitive part of TG-induced Ca^2+^ ER store release in RAW 264.7 cells (Supplemental Fig. S3A). Moreover, IgG-induced phagocytosis (Supplemental Fig. S3B), C5a-elicited chemotaxis (Supplemental Fig. S3C) and LPS-induced secretion of TNFα (Supplemental Fig. S3D) were suppressed by BTP2. The inhibitory effect of BTP2 on cytokine production was dependent on the presence of STIM2. Silencing of STIM2 and treatment with BTP2 each reduced LPS-induced TNFα (Supplemental Fig. S3E) and NF-κB activity (Supplemental Fig. S3F) in RAW 264.7 cells which, however, was not synergistic when *Stim2* KD cells were treated with BTP2. We also observed that Orai1 was not required for STIM2-regulated function. In contrast to *Stim2*^−/−^ PMs and *Stim2* KD RAW 264.7 cells, LPS-induced secretion of TNFα was neither reduced in *Orai1* KD cells (Supplemental Fig. 4A, B) nor in *Orai1*^−/−^ PMs (Supplemental Fig. S4C, D). BTP2 inhibition of TNFα was equally effective in Orai1-sufficient and Orai1-deficient PM cells (Supplemental Fig. S4D).

### BTP2 limits lethality in AIHA and LPS-induced sepsis

**Figure 9:**
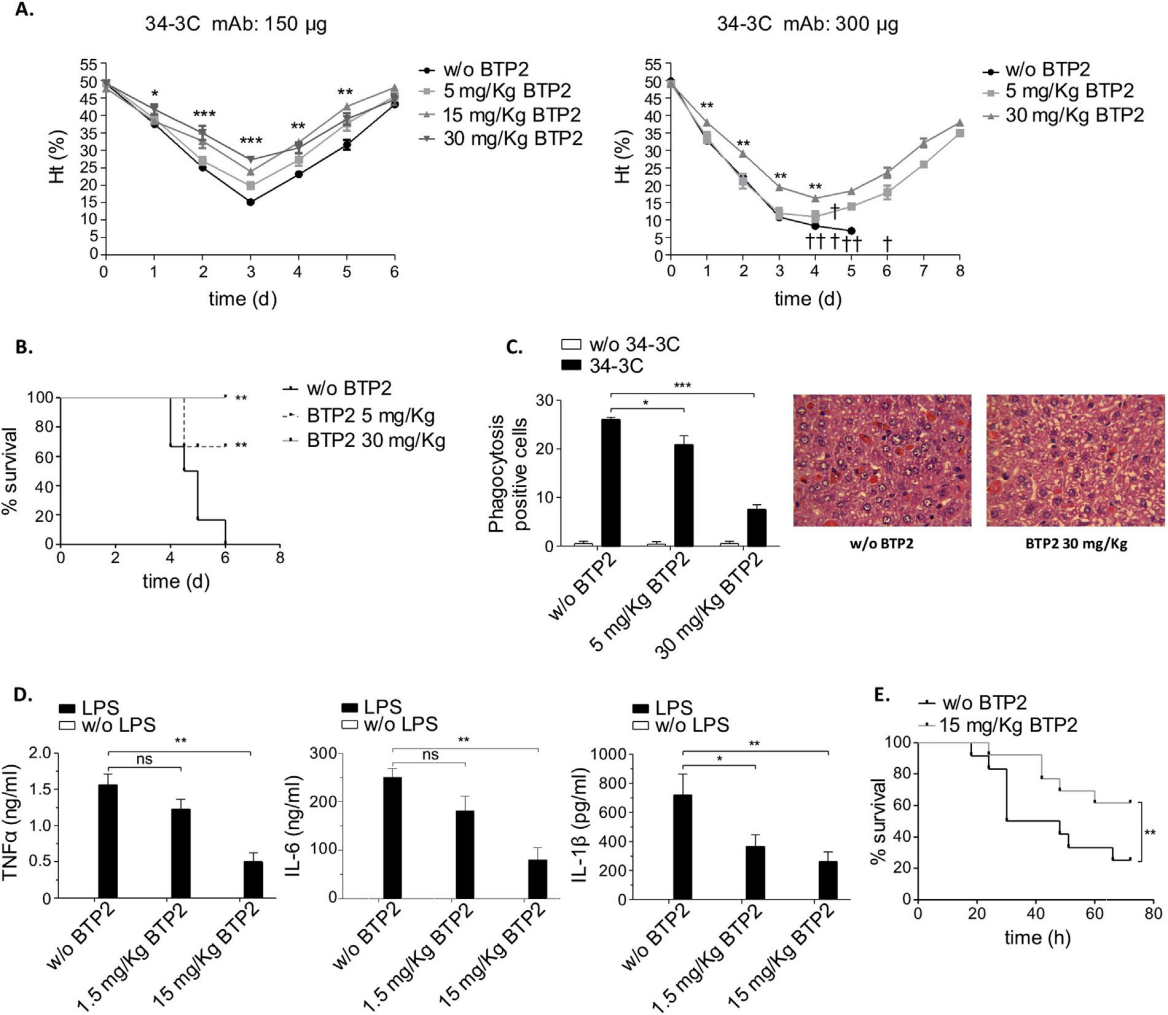
BTP2 limits severity and mortality in experimental AIHA and LPS-induced sepsis. (A, B) Transient to fatal anemia was induced by injection of 150 and 300 μg of the pathogenic 34-3C IgG2a mAb in mice treated with or without (w/o) the indicated amount of BTP2, and daily hematocrit was assessed. Death events are shown by a cross in (A), and survival rates are shown in (B). (C) Liver H&E sections from 2 days anemic mice were evaluated both quantitatively (left) and qualitatively (right) for erythrophagocytosis. The results were obtained from five to six mice in each group (*P < 0.05; **P < 0.01; ***P < 0.001). (D) BTP2 (1.5–15 mg/kg)-treated mice received 10 mg/kg LPS and serum levels of TNFα, IL-6, and IL-1β were examined at 2 h. The results shown are mean ± SEM of nine mice per group (ns, not significant; *P < 0.05; **P < 0.01). (E) The indicated mice treated or not with 15 mg/kg BTP2 received a dose of 30 mg/kg of LPS and percentage survival (*n* = 12–13 per group) is shown (**P < 0.01).

To examine the therapeutic potential of Ca^2+^-inhibition for suppression of exacerbated immune responses in vivo, we finally tested BTP2 in sublethal and lethal forms of AIHA and sepsis. In the model of AIHA, C57Bl/6 mice were injected with 150 and 300 μg of the pathogenic 34-3C IgG2a and received increasing BTP2 concentrations (5–30 mg/kg per os) or carrier along with 34-3C mAb challenge. Similar to the patterns observed in STIM1-deficiency 37, mice treated with 30 mg/kg of BTP2 displayed significant reduction/protection with respect to Ht levels and lethality (Fig. 9A, B). Moreover, BTP2 not only decreased the transient drop in Ht in a dose-dependent manner, but also reduced in vivo erythrophagocytosis (Fig. 9C). Macrophage recruitment in Thg-induced peritonitis (15.52 ± 1.68 × 10^6^ cells, *n* = 8) was also found to be significantly decreased after BTP2 treatment (8.44 ± 1.93 × 10^6^ cells, *n* = 9, *P* = 0.0023). These data suggest that BTP2 can interfere with systemic inflammatory responses that involve activation of STIM1 and STIM2. So, we next evaluated whether BTP2 also limits mortality in LPS-induced fatal sepsis. Administration of 15 mg/kg of BTP2 markedly reduced the elevated TNFa, IL-6, and IL-1β cytokine response after 10 mg/kg LPS challenge (Fig. 9D) and led to significant improvement of survival in mice treated with a lethal dose of LPS (30 mg/kg i.p.) (Fig. 9E). Together, the results show that BTP2 is effective in reducing exacerbated lethal responses in AIHA as well as sepsis.

## Discussion

STIM1 is a crucial activator of store-operated Ca^2+^ entry and the function of CRAC channels in immune cells 29,41. In the absence of STIM1, T, and B cells showed defective SOCE in response to TCR and BCR stimulation 32,33. STIM1-mediated SOCE is also a central mechanism of Ca^2+^ entry downstream of FcϵRI and FcγR activation in mast cells and macrophages 37,42. STIM2, a related homolog of STIM1, may additionally activate SOCE 43. However, the loss of STIM2 in T and B cells had a smaller negative effect on SOC influx than STIM1 32,33. STIM2 is also suggested to act as feedback regulator that stabilizes ER Ca^2+^ levels 34, while STIM1 seems to play a less critical but variable role 34,37,42.

Here, we have examined the role of STIM2 in relation to STIM1 in various Ca^2+^-dependent effector functions of macrophages. We used STIM protein-deficient mice, RNA-mediated interference, and Ca^2+^ chelation approaches to demonstrate that STIM2 is a critical regulator of GPCR-mediated cell migration and TLR4-dependent cytokine production, two macrophage responses shown here to be compromised by inhibition of intracellular Ca^2+^ but not to depend on outside Ca^2+^, or STIM1. We also noted reduced Ca^2+^ release from intracellular stores in the absence of STIM2. This was also reflected by *Stim2* KD cells. No alteration in the expression levels of STIM1 was detected in the absence of STIM2 (Supplemental Fig. S1), suggesting distinct requirements for STIM1 and STIM2 in the regulation of certain Ca^2+^ signals. Although further investigation is needed to understand the molecular basis of the functional difference between STIM1 and STIM2, our findings clearly indicate that STIM2 is the essential STIM-protein in triggering chemotactic cell migration and TLR4-activation, two processes that also contribute to inflammatory reactions in mouse models of peritonitis and LPS-induced sepsis. It is important to note, however, that it is at present not clear whether macrophages alone or together with other cell types, most notably neutrophils, mediate the STIM2-specific response in vivo.

Despite identification of STIM1 as a signal mediator of FcγR-mediated functions, the mechanisms by which Ca^2+^ is mobilized and controls phagocytosis are still not fully resolved 37. For example, residual FcγR-induced Ca^2+^ influx previously detected in STIM1-deficient macrophages might be explained by STIM2-regulated SOCE 44. Indeed, STIM2-deficient macrophages showed reduced FcγR-dependent Ca^2+^ influx as well as SOCE. However, these defects were more pronounced in STIM1 as compared to STIM2 deficiency, which is similar to previous analyses of STIM1- and STIM2-deficient T and B cells 32,33,45. In addition, FcγR-mediated phagocytosis, which depends on the availability of Ca^2+^ from both the outside and internal stores, is almost abolished or reduced in the absence of STIM1 and STIM2, respectively. These data indicate a cooperative function of STIM1 and STIM2 for efficient SOCE and FcγR-mediated phagocytosis in macrophages.

IgG autoantibody-mediated phagocytosis contributes many autoimmune conditions and is specifically causal in AIHA. In line with the defects in FcγR-mediated Ca^2+^-influx and phagocytosis, *Stim1*^−/−^ chimeric mice were significantly protected from IgG-induced elimination of red blood cells in anemia (Fig. 3) 37. Surprisingly however, IgG-induced in vivo erythrophagocytosis was STIM2-independent, contrasting the situation in vitro. A major difference might be the contribution of complement receptor signals. Recently, it was reported that the production of C5a in the liver positively regulates FcγRs and phagocytosis in AIHA autoimmune disease 14. We here found that the phagocytosis promoting effect of C5a—either in recombinant form or used as biological fluid from anemic mice—strictly depends on the presence of STIM1, whereas C5a was sufficient to counteract the basal defects of phagocytosis in *Stim2*^−/−^ macrophages. Thus, while both STIM1 and STIM2 mediate phagocytosis in response to FcγR-activated Ca^2+^ increase in vitro, the results of FcγR activation in conjunction with C5aR suggest STIM1 as the main signal mediator of the regulatory loop of C5a and FcγRs in AIHA in vivo.

BTP2 is considered a calcium influx inhibitor affecting the function of STIM1 and several different calcium channels, especially CRAC and transient receptor potential channels, in various cell types 39–41,46–49. Moreover, BTP2 has been successfully used to reduce exacerbated immune responses in animal models of allergy 40, graft versus host disease 39, and vascular inflammation 48. These data combined with our finding that BTP2 is a potent inhibitor of FcγR-induced activation and SOCE in macrophages suggest that STIM1 may be inhibited by BTP2. In accordance, BTP2 prevents lethality in severe AIHA. Interestingly however, we also observed significant reduction of migration and Ca^2+^ ER store release in BTP2-treated cells, indicating an immunosuppressive function of BTP2 that may affect STIM1 as well as STIM2-regulated functions. As shown for LPS-induced NF-κB activation and TNFα production, the inhibitory effect of BTP2 requires the presence of STIM2, indicating that BTP2 can influence STIM proteins (in this case STIM2) most likely through direct inhibition.

Here, we identified STIM2 as an important mediator of LPS-induced cytokine production in macrophages. In the absence of STIM2, the increase of circulating TNFα and other cytokines is reduced in LPS-challenged mice. Low serum cytokine levels are also seen in BTP2-treated mice. Furthermore, BTP2 resulted in enhanced survival of mice receiving a high dose of LPS. The selective absence of STIM2 in hematopoietic cells also results in an improved survival after LPS challenge. The results concerning chemotaxis of macrophages revealed that STIM2 is required for CCL2 and C5a-dependent migration in vitro and Thg-induced macrophage recruitment in vivo. Since these reactions are not largely dependent on STIM1 but are reduced in BTP2-treated mice, STIM2 may be a possible target through which BTP2 suppresses cell migration and provides protection against cytokine-mediated effects in sepsis.

Our work extends previous reports that describe a dominant contribution of STIM1 in SOCE-mediated immune cell activation 32,33,37,42. The results presented here provide evidence that STIM2 serves as critical and selective regulator of TLR4-activated cytokine production and GPCR-mediated cell migration. We also note that loss of STIM2 correlates with reduced functions of SOCE and FcγR-mediated phagocytosis, indicating that STIM2 may contribute to some degree to cell destructive events in autoimmune diseases. In the experimental model of AIHA, however, autoantibody-induced cellular destruction occurs via multiple FcγR and C5aR signals favoring a dominant contribution of STIM1. The pyrazole derivative BTP2 is effective to block several cell responses related to STIM activation and this may account for the improved capacity of mice to withstand lethal challenges with hemolysis-inducing antibodies and LPS. In summary, our study suggests the potential of Ca^2+^-inhibition for the treatment of septic inflammation and autoimmune injury and identifies a STIM-isoform-specific role for STIM2 in certain macrophage effector responses that do not strictly rely on extracellular Ca^2+^ influx. Future work has to elucidate the molecular basis of the alternate and cooperative functions of the STIM1/2 isoforms. For example, it remains to be investigated whether structural differences of STIM proteins in the interaction with plasma membrane Ca^2+^ channels and/or ER-resident Ca^2+^ pumps determine their distinct behavior in macrophages and maybe other innate immune effector cells.

## Materials and Methods

### Mice

The generation and phenotypic characterization of *Stim1*^−/−^ and *Stim2*^−/−^ mice has been described previously 50,51. STIM1 deficiency is associated with approximately 70% perinatal lethality and surviving animals display pronounced growth retardation and a maximal life span of 4–6 weeks 50. Therefore, 5–6-week-old C57Bl/6 female mice were lethally irradiated with a single dose of 10 Gy and transplanted with bone marrow from WT or *Stim1*^−/−^ mice derived from the same litters as described 37. C57Bl/6 mice were purchased from Charles River Laboratories. Genotyping of *Stim2*^−/−^ mice was performed using the following primer pairs: *Stim2 *wt allele forward primer 5′-CCCATATGTAGATGTGTTCAG-3′; reverse primer 5′-GAGTGTTGTTCCCTTCACAT-3′; *Stim2* knockout allele forward primer 5′-TTATCGATGAGCGTGGTGGTTATGC-3′; reverse primer 5′-GCGCGTACATCGGGCAAATAATATC-3′. All mice (*Stim1*^−/−^ chimeric, *Stim2*^−/−^, and their matched littermate controls) were used at 8–14 week of age. Animal experiments were conducted in accordance with current laws in combination with the regulations of the local authorities.

### Macrophage preparation and characterization

Peritoneal macrophages (PMs) were collected after flushing out the peritoneal cavity of mice with RPMI, without any supplements. Cells were washed twice and suspended in RPM1 1640 medium containing 10% FCS. The PM cells were allowed to adhere for 4 h at a density of 3 × 10^6^ cells/well of 6-well plate cell culture dishes (Costar, Munich, Germany), followed by the removal of nonadherent cells, and were then used in various macrophage activation assays as described below. *Stim1*^−/−^ and *Stim2*^−/−^ F4/80- and Mac1-positive (> 95%) PM were microscopically indistinguishable from wild-type controls, exhibited unaltered mRNA expression of Orai1, Orai2, and Orai3 (Figs. 3B, 7B) and showed normal adhesion and IgG-MRBC rosette formation (data not shown).

### Macrophage Ca^2+^ depletion/chelation and STIM inhibition

PMs or RAW264.7 cells were incubated with Bapta/AM (Calbiochem, Darmstadt, Germany) at 50 µM for 30 min. Cells were subsequently washed and given 30 min as esterification time. This was followed by two final washing steps. EGTA (Sigma–Aldrich, Munich, Germany) was applied in assay medium at 2 mM immediately prior to cell stimulation. The bistrifluoromethyl pyrazole (BTP) derivative BTP2 (Sigma–Aldrich) was used at 20 μM for 1 h prior to stimulation.

### Generation of macrophage *Stim1* and *Stim2* knockdown (KD) cells

Up to five distinct STIM1 and STIM2 short hairpin RNA (shRNA) and mock control (no and scramble shRNA) pLKO.1-puro and pRFP-C-RS plasmids (Sigma; Origene) were used for transfection of RAW264.7 cells. All transfections were performed with Lipofectamine 2000 following the manufacturer's protocol (Invitrogen). Stably transfected cells were selected with 3 µg/mL puromycin and expression of *Stim1* and *Stim2* transcripts were evaluated with SYBR Green real-time qPCR using the following primer pairs: *Stim1* forward: 5′-AGCTGATGGATGACGATGG-3′, *Stim1* reverse: 5′-TTGAGGTCTTCCCTTAGG AACTC-3′, *Stim2* forward: AACCAACAACCCCCAACACC-3′, *Stim2* reverse: 5′-ATCAGCGACCGA-3′. shRNA-transfected RAW264.7 cell clones with high efficiency of silenced *Stim1* and *Stim2* mRNA expression were chosen and these stable KD cells were further validated by immunoblot analysis. STIM1 deficiency was confirmed using an anti-STIM1 mAb from BD Transduction (GOK/Stim1, clone number 44) as described 37. Protein analysis of STIM2 was performed using an anti-STIM2 Ab (Sigma–Aldrich). The relative expression of STIM1 and STIM2 for each sample was determined densitometrically and normalized to the expression of β-tubulin (detected with rabbit anti-β-tubulin; Abcam, Cambridge, UK) in the same sample.

### Analysis of NF-κB activity in *Stim2* KD cells

*Stim2* KD and mock control cells 5 × 10^5^ were transfected with 1 μg of the pGL4.32 [*luc2P*/NF-κB-RE/Hygro] and 0.1 μg of the reference *Renilla* luciferase plasmid, which was used as a transfection control. The cells were recovered after 24 h, cultured for 24 h in RPM1 1640 medium containing 1% FCS. and treated with 100 ng/mL LPS for 3 h or left untreated. To measure luciferase activity we used the Dual-Luciferase reporter assay system (Promega). The firefly luciferase activity was normalized to *Renilla* luciferase activity and the activity of stimulated cells to the one of untreated cells to yield the relative promoter activity driven by NF-κB.

### Functional analysis of macrophage effector responses

#### IgG-induced phagocytosis

Freshly isolated mouse RBCs (MRBCs) from C57Bl/6 mice were washed and processed for IgG opsonization. Hereby, 100 µL of MRBCs were incubated with an equal volume of PBS supplemented with 50 µg/mL anti-MRBC IgG2a for 1 h. MRBCs were then washed and suspended at the original volume. PM cells were exposed to a 4% MRBC solution in RPMI 1640 medium/10% FCS for 3 h. In some experiments, PM cells were additionally incubated with C5a (50 ng/mL) or AIHA-derived liver supernatants. Non-ingested RBCs were removed by hypotonic lysis, PMs were washed with PBS, fixed with 4% paraformaldehyde, stained, and phagocytosis was determined by light microscopy. A slightly modified assay was used for the RAW264.7 macrophage cell line, where cells were first allowed to adhere overnight in RPMI 1640 medium/1% FCS at a density of 1.5 × 10^5^ cells/well before washing and exposure to a 4% MRBC buffer in RPMI 1640 medium/10% FCS for 90 min.

#### LPS-induced cytokine production

PM cells (2 × 10^5^/well) were incubated for 24 h with 100 ng/mL LPS from *Escherichia coli* O127:B8 (Sigma–Aldrich) in 96-well plates (Costar). LPS-induced activation was analyzed for cytokine release into culture supernatants by TNFα and IL-6-specific ELISA kits (R&D Systems, Wiesbaden, Germany). Measurements were performed according to manufacturer's instruction.

#### C5a/CCL2-induced chemotactic migration

PMs or RAW264.7 macrophages 5 × 10^5^ in 100 μl RPMI 1640 medium/0.1% BSA (fatty acid free) were placed into the insert of a Transwell chemotaxis chamber (8-µm pore diameter; Greiner, Frickenhausen, Germany), and the bottom well was filled with 500 μl of RPMI 1640 medium/0.1% BSA (fatty acid free) or the same medium supplemented with C5a (50 ng/mL) or CCL2 (100 ng/mL). Inserts were transferred to the lower chambers and incubated at 37°C for 4 h. For quantification of chemotaxis, Transwell filters were fixed in methanol, stained with May–Grünwald–Giemsa. Cells on the upper side of the filter were removed and filters were mounted on a glass slide. Migrated macrophages that attached to the filter were counted by light microscopy as described 52.

#### SOCE and FcγR-induced Ca^2+^activation

PM cells (10^6^/mL) in HBSS buffer (120 mM NaCl, 5 mM KCl, 1 mM MgCl_2_, 20 mM Hepes pH 7.4, 1 mM CaCl_2,_ 10 mM Glucose, 0,5% BSA) were loaded with Fura-2/AM (5 µΜ; Calbiochem) in the presence of pluronic F-127 (0.2 µg/mL; Sigma–Aldrich) for 30 min at 37°C. After labeling, the cells were washed twice and activated with 2 µM of the sarcoplasmatic/endoplasmatic reticulum Ca^2+^ ATPase pump inhibitor Thapsigargin (TG; Invitrogen). The analyses of TG-induced Ca^2+^ ER store release and subsequent SOCE were performed in HBSS buffer containing 2 mM EGTA or 1 mM Ca^2+^, respectively, and fluorescence was measured with an LS 50 spectrofluorimeter (PerkinElmer, Waltham, USA). Excitation was alternated at 340 and 380 nm, and emission was measured at 509 nm. Each measurement was calibrated with Triton X-100 and EGTA and intracellular concentration of calcium was calculated according to Grynkiewicz et al. 53. To measure FcγR-induced activation of intracellular Ca^2+^, cells were first incubated with 10 μg/mL of rat anti-FcγRIII (clone 275003) 15 and 10 μg/mL of Armenian hamster anti-FcγRIV (clone 9E9) 15,54 IgG mAbs followed by 20 μg/mL of either rabbit anti-rat IgG (R&D Systems) or rabbit anti-Armenian hamster IgG (Abcam) for cross-linking of FcγRIII or FcγRIV, respectively.

### Mouse models of inflammation

#### Experimental sepsis

Mice were injected i.p. with a dose of 10 mg/kg LPS/5% FBS (Sigma–Aldrich). Two hours post injection, blood was collected from the retro-orbital plexus. Serum was collected after blood was left at RT for 45 min and spun at 3000 rpm for 10 min. Serum cytokine levels were assayed by TNFα-, IL-6-, and IL-1β-specific ELISA kits (R&D Systems). In BTP2-inhibition experiments, C57Bl/6 mice received single doses of 5 and 15 mg/kg BTP2 i.p. or vehicle control 1 h before LPS challenge (10 and 30 mg/kg), survival was monitored, and serum cytokines were determined by ELISA.

#### Thg-induced peritonitis

Peritoneal recruitment of leukocytes was induced using Thg medium (PBS/4% brewer thioglycollate; BD-BBL, Heidelberg, Germany). Thg medium was initially produced at 8%, left to mature for 4 weeks, and diluted to 4% with 2× PBS immediately before i.p. injection. In experiments evaluating the effect of BTP2, mice received together with Thg a daily dose of 30 mg/kg BTP2 or vehicle control per os—intragastric, via gavage for 3 days. On day 4 post Thg injection, mice were killed, and the peritoneal cavity was lavaged two times with 5 mL of PBS/5mM EDTA. Total cell count of collected fluids was determined in a hemocytometer (Neubauer Zählkammer, Gehrden, Germany). For quantification of macrophage influx, differential cell counts were performed on cytospins (10 min at 50 ×* g*).

#### Experimental AIHA

Mouse anti-MRBC IgG2a monoclonal autoantibody 34-3C and its IgG2b genetic variant 15,55 were purified from tissue-culture supernatants by protein G affinity chromatography. Mice were injected i.p. with 150–300 μg of the pathogenic mAbs, survival was monitored, and daily hematocrit (Ht) was determined with heparinized microhematocrit capillary tubes using blood samples obtained from the retro-orbital plexus. In BTP2-inhibition experiments, mice further received daily doses of 5, 15, and 30 mg/kg BTP2 or vehicle control per os for 3 days. H&E-stained formalin-fixed sections of liver were prepared from mice killed at days 0 and 2 after 34-3C mAb treatment and examined for histopathologic changes. To produce liver supernatants (from day 2 of AIHA), livers were perfused, excised, minced into small pieces, and treated with PBS *+* 0.025% Collagenase IV (Sigma–Aldrich). This was followed by passages through a stainless steel gauze (mesh, 500 and 100 µm), so that single cell suspensions are obtained. Hepatocytes and the nonparenchymal cell fraction were removed by centrifugation and the obtained supernatant was stored at −80 °C for later use as described 14.

### Statistics

Statistical analysis was performed using the Prism 5 statistical software package (GraphPad Software). Comparisons between groups were analyzed with 2-tailed Student's *t*-test. Differences in means among multiple groups were analyzed using one-way ANOVA with the Tukey's post-test.

## Supporting Information

Figure S1. Generation of macrophage *Stim1* and *Stim2* KD cells. **Figure S2.** Different effects of intracellular vs. extracellular Ca^2+^ chelation/inhibition on RAW264.7 cell mediated functions. **Figure S3.** Defective TG-induced Ca^2+^ ER store release, SOCE, phagocytosis, chemotaxis, and TLR4 activation in BTP2-treated RAW264.7 macrophages. **Figure S4.** LPS-induced TNFα release occurs normally in *Orai1* KD cells and *Orai1*^-/-^ PM.
