# Supplementary material for: Cooperative and alternate functions for STIM1 and STIM2 in macrophage activation and in the context of inflammation
Source: Immun Inflamm Dis. 2015 May 12;3(3):154–70. doi: 10.1002/iid3.56 (PMC4578517; doi:10.1002/iid3.56)
Supplement: Figure S1. — Generation of macrophage Stim1 and Stim2 KD cells. Figure S2. Different effects of intracellular vs. extracellular Ca2+ chelation/inhibition on RAW264.7 cell mediated functions. Figure S3. Defective TG-induced Ca2+ ER store release, SOCE, phagocytosis, chemotaxis, and TLR4 activation in BTP2-treated RAW264.7 macrophages. Figure S4. LPS-induced TNFα release occurs normally in Orai1 KD cells and Orai1-/- PM. [file iid30003-0154-sd1.pdf]

Supplementary Figures

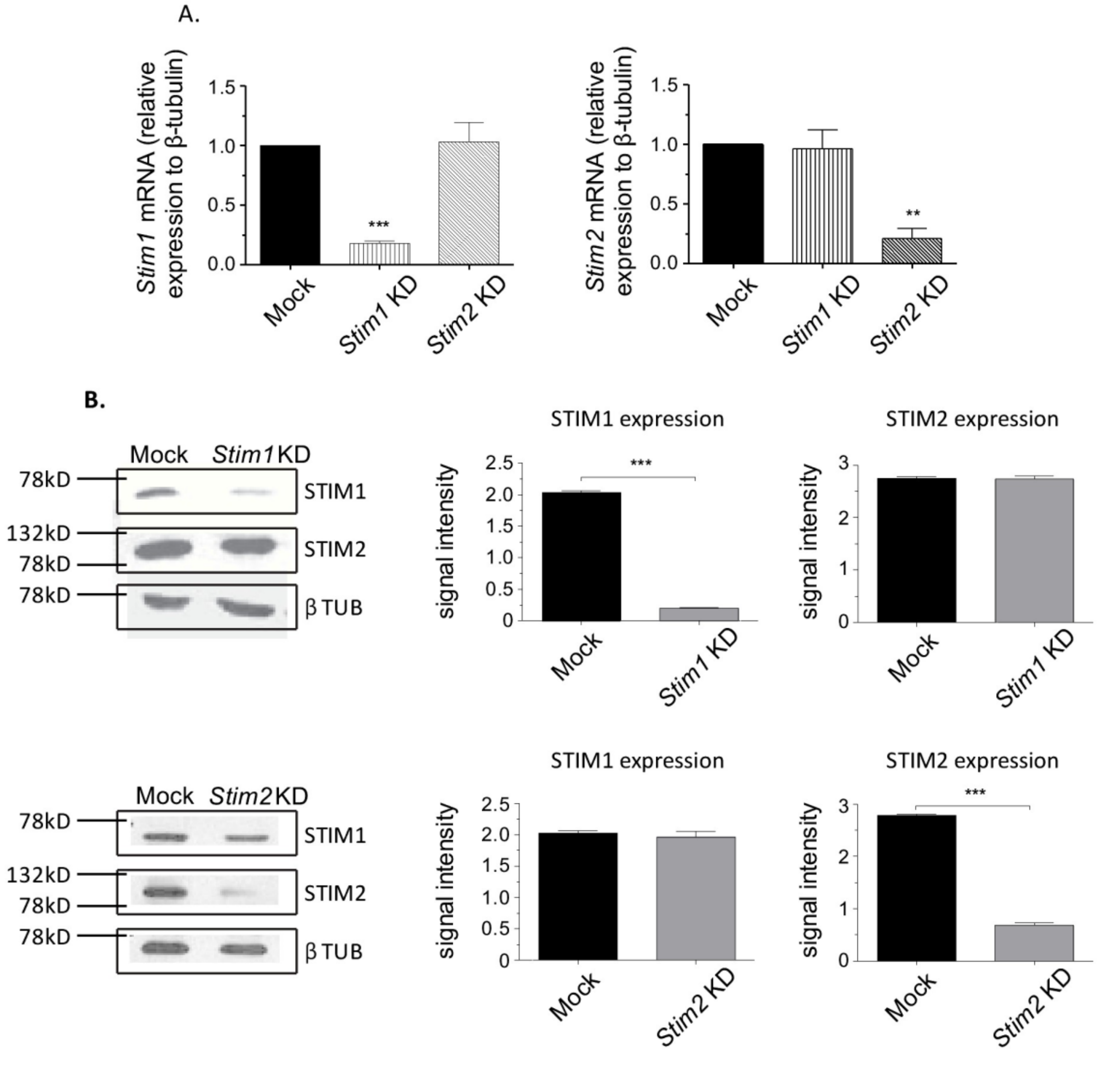

Suppl. Figure 1: Generation of macrophage *Stim1* and *Stim2* KD cells.

(**A)** Real-time RT-PCR (ΔΔCt) analysis of *Stim1* and *Stim2* mRNA following treatment of RAW264.7 cells with shRNA against STIM1 or STIM2. Results of qPCR-selected *Stim1* and *Stim2* KD RAW264.7 cell clones with high shRNA silencing efficiency are shown. Data are expressed as mean relative *Stim* to ß-tubulin mRNA ± SEM of n=3 independent experiments (***$P < 0.001$). (**B**) Immunoblot analysis of STIM1 and STIM2 expression in mock-transfected and *Stim1* and *Stim2* KD RAW264.7 cells (upper part: *Stim1* KD analysis; lower part: *Stim2* KD analysis); ß-tubulin served as a loading control. Left panels: representative blots. Middle and right panels: densitometric analysis of individual blots. The results shown are the mean signal intensities (± SEM) for STIM1 and STIM2, each normalized to the signal intensity of the ß-tubulin signal of the same sample (arbitrarily defined as 1.0) (***$P < 0.001$).

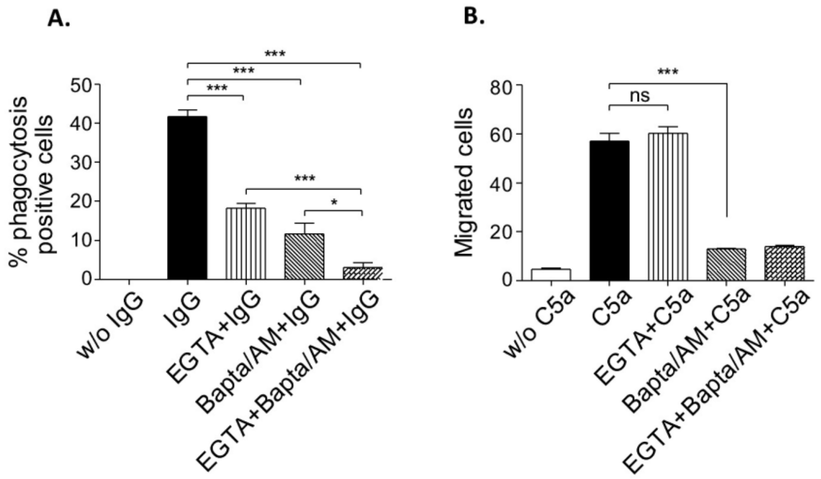

Suppl. Figure 2: Different effects of intracellular vs. extracellular Ca^{2+} chelation/inhibition on RAW264.7 cell mediated functions.

RAW264.7 cells were cultured in the presence of Ca^{2+}-specific chelators (EGTA, or Bapta/AM) or a combination of both (EGTA + Bapta/AM) and assayed for (**A**) IgG-induced phagocytosis (IgG), and (**B**) C5a-induced chemotactic migration (C5a). (**A**) RAW264.7 cells were incubated with uncoated (w/o IgG) or IgG-coated (IgG) MRBC for 90 min. at 37°C followed by the lysis of extracellular MRBC. The percentage of positive cells that ingested more than 1 MRBC was assessed microscopically. (**B**) RAW264.7 cells were stimulated for 4 hours with or without (w/o) 50 ng/ml of C5a and migrated cells were determined by Transwell migration assay. All results are expressed as mean ± SEM of 4 independent experiments (ns; *P < 0.05; ***P < 0.001).

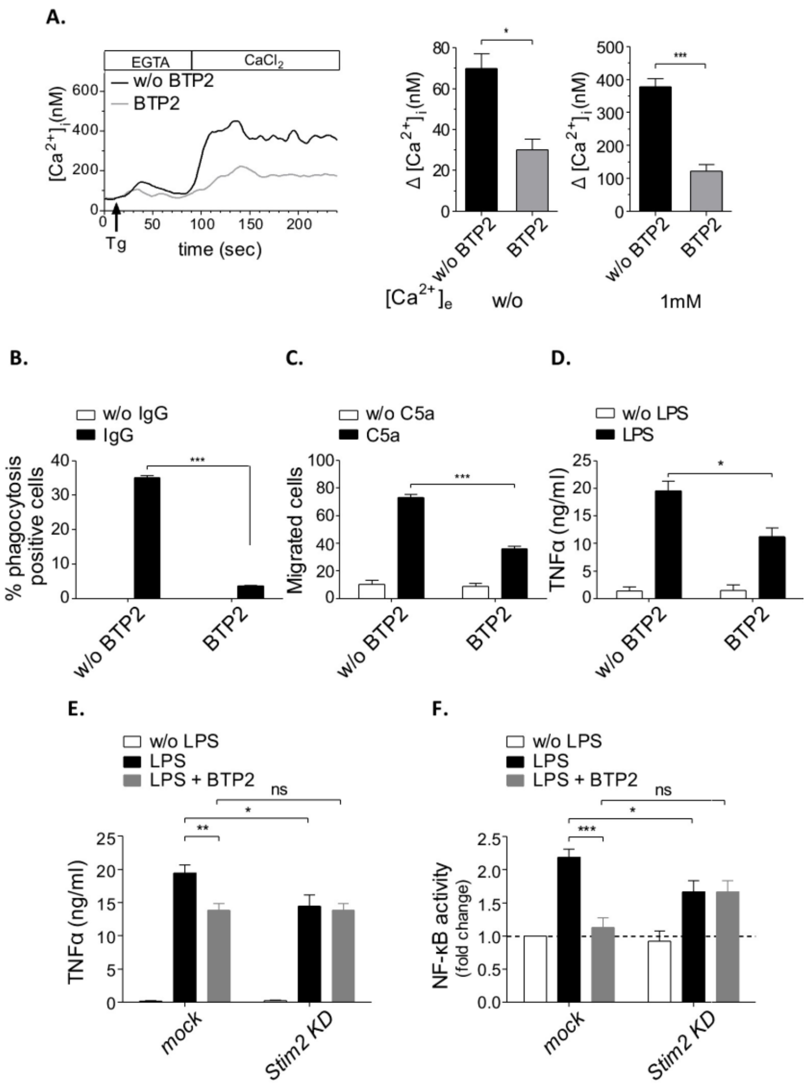

Suppl. Figure 3: Defective TG-induced Ca^{2+} ER store release, SOCE, phagocytosis, chemotaxis, and TLR4 activation in BTP2-treated RAW264.7 macrophages.

(A) RAW264.7 cells treated or not with BTP2 were loaded with Fura2/AM, stimulated with 2 μM TG in EGTA-containing buffer (w/o $[Ca^{2+}]_e$) followed by the addition of $CaCl_2$ (1 mM $[Ca^{2+}]_e$) and monitoring of $[Ca^{2+}]_i$. Representative measurements (left panel) and maximal ($\Delta[Ca^{2+}]_i$ ± SEM) values (n=4 per group) in the absence (w/o $[Ca^{2+}]_e$) (middle panel) and presence (1 mM $[Ca^{2+}]_e$) of extracellular Ca^{2+} (right panel) are shown (*P < 0.05; ***P < 0.001). Both TG-induced Ca^{2+} ER store release and SOCE are blocked by BTP2. **(B)** BTP2-treated cells were incubated with uncoated (w/o IgG) or IgG-coated MRBCs and the percentage of phagocytosis was assessed. **(C)** C5a-induced chemotaxis was determined by Transwell migration assay. **(D)** LPS-induced secretion of TNFα was analyzed by ELISA. **(E)** *Stim2* KD RAW264.7 cells and mock-transfected controls were treated with BTP2 or left untreated, stimulated with 100 ng/ml LPS and analyzed 24 hours later for TNFα release by ELISA. **(F)** *Stim2* KD and mock control cells were transfected with the pGL4.32 [*luc2P*/NF-κB-RE/Hygro]. Relative NF-κB activity 3 hours after LPS stimulation in BTP2-treated or untreated cells is depicted. **(B-F)** Results are expressed as mean ± SEM of 3 independent experiments (*P < 0.05; **P < 0.01; ***P < 0.001).

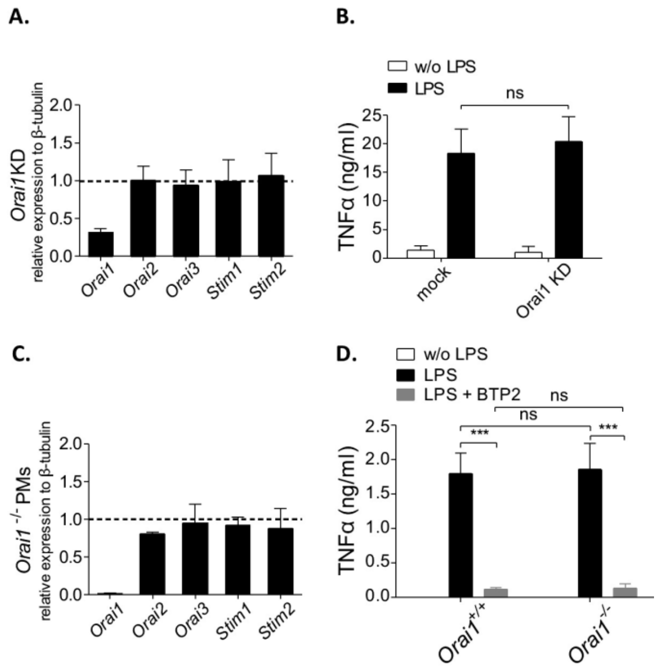

Suppl. Figure 4: LPS-induced TNFα release occurs normally in *Orai1* KD cells and *Orai1*$^{-/-}$ PM.

(A) Real-time RT-PCR (ΔΔCt) analysis of *Orai* / *Stim* mRNA following treatment of RAW264.7 cells with shRNA against Orai1. **(B)** *Orai1* KD cells were stimulated with 100 ng/ml LPS and analyzed at 24 hours for the release of TNFα by ELISA. **(C)** Real-time RT-PCR (ΔΔCt) analysis of *Orai* / *Stim* mRNA in *Orai1*$^{-/-}$ PM cells (the dashed line indicates wild-type expression in *Orai1*$^{+/+}$PM cells). **(D)** *Orai1*$^{-/-}$ and *Orai1*$^{+/+}$ PM cells were treated with BTP2 or left untreated and stimulated with 100 ng/ml LPS and analyzed at 24 hours for the release of TNFα by ELISA. **(A-D)** Results are expressed as mean ± SEM of 3 independent experiments (***P < 0.001; ns = not significant).
